# Surface Chemistry of Cytosporone-B Incorporated in Models for Microbial Biomembranes as Langmuir Monolayers

**DOI:** 10.1021/acs.langmuir.4c01575

**Published:** 2024-07-15

**Authors:** Guilherme Nuñez Jaroque, Augusto Leonardo dos Santos, Patricia Sartorelli, Luciano Caseli

**Affiliations:** Department of Chemistry, Institute of Environmental, Chemical and Pharmaceutical Sciences, Federal University of São Paulo (Unifesp), São Paulo, Diadema 04021-001, Brazil

## Abstract

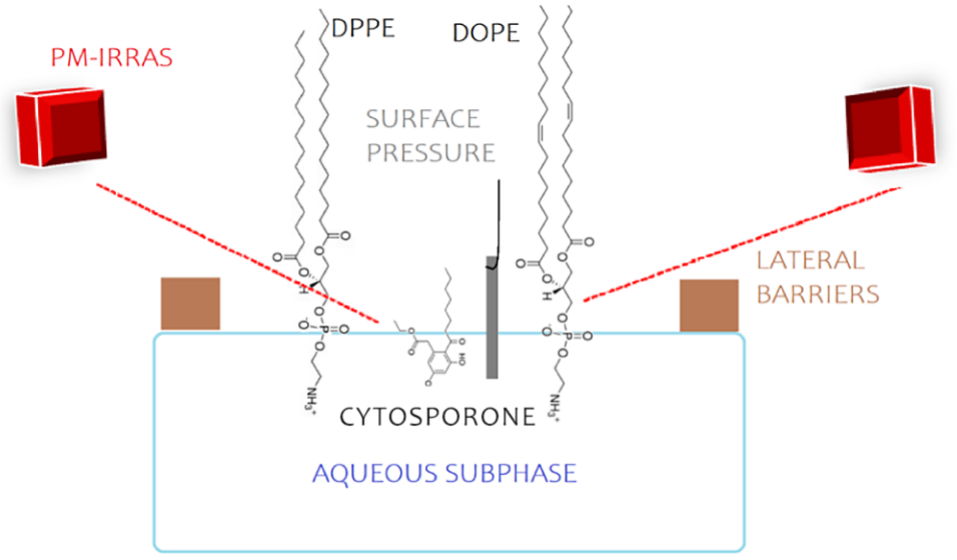

Cytosporone-B, a polyketide renowned for its antimicrobial properties, was integrated into Langmuir monolayers composed of dipalmitoylphosphoethanolamine (DPPE) and dioleoylphosphoethanolamine (DOPE) lipids, effectively emulating microbial cytoplasmic membranes. This compound exhibited an expansive influence on DPPE monolayers while inducing condensation in DOPE monolayers. This led to a notable reduction in the compressibility modulus for both lipids, with a more pronounced effect observed for DPPE. The heightened destabilization observed in DOPE monolayers subjected to biologically relevant pressures was particularly noteworthy, as evidenced by surface pressure–time curves at constant area. In-depth analysis using infrared spectroscopy at the air–water interface unveiled alterations in the alkyl chains of the lipids induced by cytosporone-B. This was further corroborated by surface potential measurements, indicating a heightened tilt in the acyl chains upon drug incorporation. Notably, these observed effects did not indicate an aggregating process induced by the drug. Overall, the distinctive impact of cytosporone-B on each lipid underscores the importance of understanding the nuanced effects of microbial drugs on membranes, whether in condensed or fluid states.

## 1 Introduction

**Figure 1:**
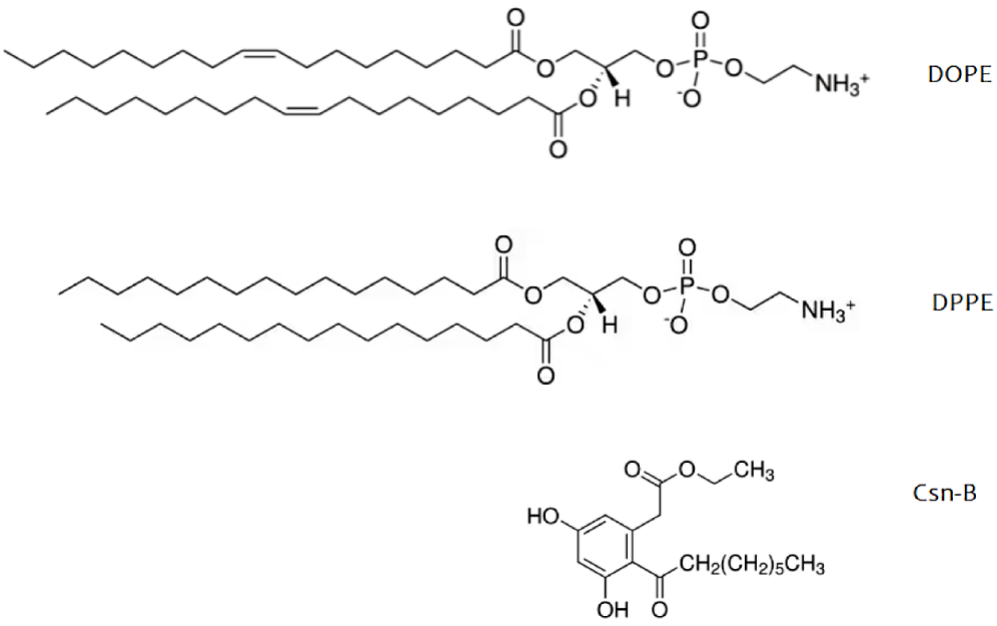
Chemical structures of DPPE, DOPE, and Csn-B.

Cytosporones, a group of natural polyketides primarily sourced from endophytic fungi,^1−4^ hold significant potential in various therapeutic applications. Among these, cytosporone B (Csn-B) (depicted in Figure 1) has garnered attention for its promising role in cancer treatment^5^ and the management of microbial and influenza viral infections.^6−8^ Its mechanisms of action as an antimicrobial agent are not as extensively studied as some other antibiotics, but research suggests several potential mechanisms,^8−10^ such as inhibition of enzymes, disruption of membrane integrity, interference with signal transduction, induction of oxidative stress, DNA damage, and disruption of biofilm formation. For the last, cytosporone might inhibit biofilm formation or disrupt preexisting biofilms, making microorganisms more susceptible to other antimicrobial agents or immune system responses. Regarding the perturbation of the microbial membrane or viral envelope, cytosporone could disrupt the integrity of microbial cell membranes. Interaction with lipid components of the cell membrane may alter membrane permeability, leading to leakage of cellular contents and eventual cell death.

These mechanisms collectively contribute to the antimicrobial activity of cytosporone. However, it’s important to note that further research is needed to fully elucidate its precise mode of action and potential applications in combating microbial infections. Understanding how cytosporones interact with lipid interfaces is of paramount interest, as these interactions could impact cell membranes and liposomes used in drug delivery strategies.

Cell membranes are intricate and multifaceted structures,^11^ making it imperative to investigate simplified models that can provide valuable insights. Artificial lipid-based membranes serve as highly effective platforms for studying the insertion of harmful substances and investigating their interactions with the lipid components found in cellular membranes. These studies provide invaluable insights into biological processes involving membranes and help to correlate these interactions with various cellular responses. One such model is lipid Langmuir monolayers.^12−17^ Notably, phospholipids with ethanolamine head groups have been employed to replicate microbial, viral, and even specific tumorigenic membranes,^18−21^ as these lipids have a higher relative abundance than healthy human cells on the external layer of the cytoplasmatic cell.^22,23^

In healthy human cells, lipids are distributed asymmetrically between the inner and outer leaflets of the cell membrane. Phosphatidylcholine (PC) primarily resides in the outer leaflet of the plasma membrane, while phosphatidylethanolamine (PE) and phosphatidylserine (PS) are predominantly found in the inner leaflet.^24,25^ Furthermore, PC and PE are frequently selected as lipid materials for simulating cell membranes and encapsulating drugs.^26,27^ These insights underscore the relevance of investigating cytosporone interactions with lipid interfaces, offering potential breakthroughs in drug delivery and therapeutic applications. As a result, PC lipids are commonly utilized to investigate toxicity in mammalian cells, whereas PE lipids serve to emulate bacterial and protozoan membranes, as well as viral envelope composition.^26,27^ Langmuir monolayers composed of PE offer a more authentic setting for examining the drug’s interaction with viruses compared to PC monolayers. This facilitates more accurate predictions regarding the drug’s efficacy against real microbes and viruses. The present paper uses PE lipids to focus on cytosporone’s interactions as a bactericidal, antiprotozoal, and antiviral compound.

As a novel compound, the existing body of literature provides limited insights into the interaction of our studied compound with various lipid types and its mechanisms of action in anticancer or antiviral contexts.^28^ However, it is crucial to investigate how this compound interfaces with cell membranes, even if it doesn’t primarily target membranes, as this interaction is vital for gaining access to the cell’s cytoplasm. Additionally, understanding its interaction with lipids is paramount, considering the potential utilization of liposomes in drug delivery.

Given these considerations, our research endeavors to shed light on cytosporone-B (Csn-B) behavior when interacting with models representing cell membranes or viral envelopes. We achieve this by employing Langmuir monolayers composed of phosphatidylethanolamine (PE) lipids (depicted in Figure 1). Specifically, we use two types of PE lipids: 1,2-dipalmitoyl-*sn*-glycero-3-phosphoethanolamine (DPPE), which forms a rigid monolayer upon compression to collapse, and 1,2-dioleoyl-*sn*-glycero-3-phosphoethanolamine (DOPE), characterized by a less elastic monolayer. These models serve as valuable tools for gaining insights into the interactions of Csn-B with lipid systems closely resembling microbial cell membranes and viral envelopes.

It is essential to underscore that our previous research has investigated the examination of established models for tumorigenic cells, explicitly focusing on palmitoyloleoylglycerophosphoserine (POPS) and dipalmitoyl glycerophosphoserine (DPPS) lipids.^29^ We observed distinctive effects upon incorporating Csn-B into DPPS and POPS monolayers at the air–water interface. Building upon this foundation, our current endeavor aims to extend this investigation to antimicrobial cells, thereby broadening the scope of our research and contributing to a deeper understanding of the interactions involving these lipids in diverse cellular contexts.

## 2 Experimental Section

### 2.1 Isolation of Csn–B

The endophytic fungus identified as *Phomopsis* sp. was isolated from *Casearia arborea* and cultivated in rice for 28 days, followed by extraction with EtOAc, generating the crude extract described in ref. (1). After that, the crude extract was partitioned between hexane and MeOH: H_2_O (9:1, v/v), and then the methanolic phase (900 mg) was then subjected to column chromatography over SiO_2_ followed by Sephadex LH-20 to give six subfractions (I to VI). Csn-B (10.2 mg) was purified from fraction IV by HPLC-DAD in RP-18, with proportions of mobile phase 10% of ACN and 90% of H_2_O from 0 to 0.5 min, increasing up to 90% of ACN in 20 min and remaining constant for 9 min, flow 1.0 mL/min. The structure of the Csn-B was fully characterized by NMR and MS data analysis and by comparison of their data with those reported in the literature.^4^

### 2.2 Surface Chemistry

DPPE and DOPE (purity of more than 99%) were purchased from Sigma-Aldrich. The lipids and Csn-B were dissolved in chloroform (Synth) to produce 0.5 mg/mL solutions.

A Langmuir trough from KSV-Nima Instruments (model mini – 220 mL of total volume) was previously cleaned with ethanol and chloroform and filled with water purified by the Milli-Q system (resistivity 18.2 MΩ·cm; pH 6.0; surface tension of 72.8 mN/m at 20 °C). DPPE and DOPE solutions were spread on the air–water interface, and 10 min waited for solvent evaporation. Selected aliquots of Csn-B were cospread with DPPE and DOPE and also left to stabilize for 10 min, determined by ideal proportions as reported previously,^31^ corresponding to 4 and 2% in molar proportion. The drug and lipids were spread from the same organic solution to guarantee homogeneity. Surface pressure–area (π–*A*) isotherms were obtained by compressing the monolayers with two barriers at a rate of 10 mm/min symmetrically. The Wilhelmy method was employed to measure the surface pressure with precision of 0.1 mN/m. Stability measurements were obtained by compressing the monolayer to 30 mN/m and following the surface pressure variation with time, keeping the film area constant. The surface potential was determined through the compression process utilizing a KSV NIMA Surface Potential Sensor. This specialized sensor gauges the potential difference above and below the film, demonstrating sensitivity to the cumulative effect of individual dipole moments. The alterations in surface potential were quantified by discerning the potential difference between the oscillating plate positioned above the monolayer and the counter electrode submerged in the subphase beneath the monolayer.

For the measurement of polarization-modulation infrared reflection-absorption spectroscopy (PM-IRRAS) spectra, the monolayers were first compressed up to a surface pressure of 30 mN/m. PM-IRRAS spectra were recorded using a KSV PMI 550 (KSV-Nima Instruments) spectrophotometer at an 80° angle of incidence relative to the interface normal, while BAM images were captured with the KSV-Nima microscope. The incident IR beam underwent modulation by a ZnSe photoelastic modulator (PEM) operating at its resonance frequency of 50 kHz, with the modulation frequency set at 1500 cm^–1^ and a retardation of λ/2. This setup allowed for separating two signals at the detector using dual-channel electronics with lock-in detection: the sum signal (or reference spectrum) and the difference signal (or surface-specific information), respectively. Simultaneous measurement of both spectra significantly reduced the effect of water vapor. The bare water surface was the background, while the film-covered surface was the sample. The PM-IRRAS signal (S) was calculated using the equation: *S* = Δ*R*/*R* = (Rp – Rs)/(Rp + Rs), where Rp and Rs represent the parallel (p) and perpendicular (s) polarized reflectances to the plane of incidence, respectively. The normalized PM-IRRAS spectrum was obtained as Δ*S* = (*S* – *S*_0_)/*S*_0_, where *S*_0_ represents the bare water surface’s signal and *S* represents the film-covered surface. Each spectrum was acquired over a total acquisition time of 10 min, resulting in 6000 interferograms per spectrum. The spectral range of the PMI 550 device spans from 800 to 4000 cm^–1^, with a resolution of 8 cm^–1^. The figures displayed raw normalized spectra before subtracting the fitting baseline and performing peak processing. Specific regions were carefully selected with caution to avoid spectra distortion when constructing the baseline. Additionally, adjustments were made to ensure spectra were horizontal when inclined.

All the experiments were carried out at a controlled temperature of 25 ± 1 °C and repeated thrice to ensure reproducibility. Representative curves, spectra, or images are shown. Room temperature was chosen following a standard protocol for lipid Langmuir film studies, which usually employed temperature (*T*) values ranging between 20 and 25 °C to attain all the 2D states of the monolayer by compressing it isothermally.

## 3 Results and Discussion

**Figure 2:**
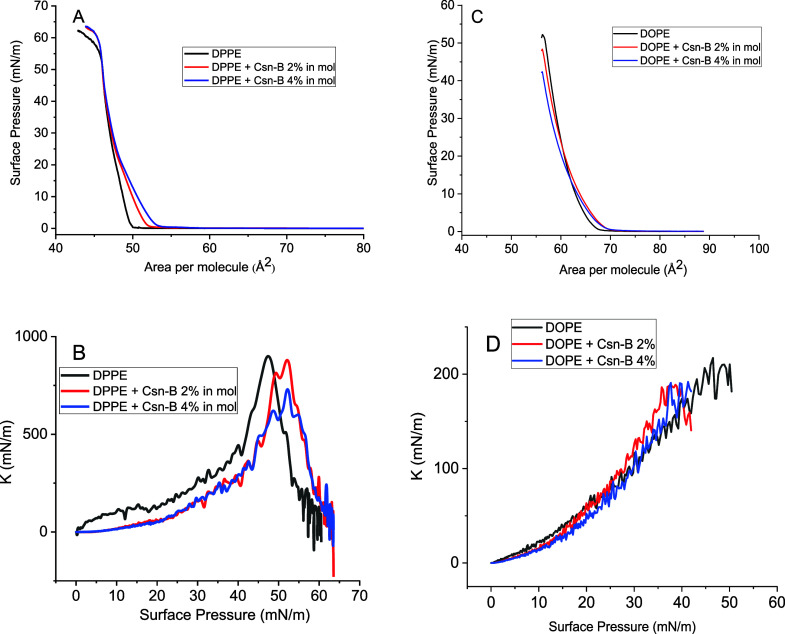
Surface pressure–area (A and C) and surface compressional modulus–surface pressure (B and D) isotherms for DPPE (A and B) and DOPE (C and D) monolayers, alone or with Csn-B. The area per molecule is related only to the lipid.

Figure 2 shows the tensiometric measurements for the lipid monolayers compressed isothermally. DPPE and DOPE present a typical curve;^30,31^ however, the unsaturations in the DOPE’s acyl chains make the monolayers less elastic, i.e., they attained 2D states with lower compressional modulus (*K*). This parameter was calculated from the surface pressure–area (π–*A*) isotherms (panels A and C) using the equation *K* = −*A* and it is shown in panels C and D.

Indeed, the behavior of DPPE and DOPE monolayers is typical, with DPPE monolayers reaching *K* values of up to 800 mN/m, indicative of a solid structure, while DOPE monolayers exhibit values as high as 200 mN/m, reflecting a liquid-condensed (LC) state.^32^ In the presence of Csn-B, DPPE monolayers undergo expansion, increasing molecular areas, signifying drug incorporation. It is crucial to clarify that the molecule area depicted on the *x*-axis exclusively pertains to the lipid, with no consideration given to the drug’s occupancy. This approach facilitates a more accurate comparison of the drug’s effects and enhances precision. This is particularly relevant because Csn-B tends to aggregate at the interface in isolation, leading to challenges in determining molecular areas as it may unevenly spread along the interface. Furthermore, we explored higher concentrations of Csn-B, approaching the limit of confirmability for the drug–lipid ratio. Given that Csn-B tends to aggregate when isolated, elevated concentrations in the mixed monolayers resulted in inconsistent effects on the isotherms. However, as surface pressures exceed 35 mN/m, the isotherms converge. While this observation could suggest that the drug does not incorporate into the monolayer at high surface pressures, other effects may explain this phenomenon. It could be attributed to cytosporone redistribution within lipid interstices, mitigating lateral repulsion or expelling from the monolayer.

Conversely, DOPE monolayers also experience expansion at higher molecular areas in the presence of Csn-B. During compression, the isotherms for the mixed monolayers intersect with the isotherm for pure DOPE at approximately 15 mN/m. Beyond this point, the drug induces more significant condensation of the mixed monolayer than the pure lipid monolayer, implying a shift toward lower molecular areas. This phenomenon may be attributed not only to the drug’s expulsion from the lateral interstices of the lipid monolayer but also to potential aggregation, possibly arising from reduced lateral repulsion due to specific interactions, such as tail–drug or head–drug interactions. Additionally, the possibility of the monolayer material being drawn into the subphase cannot be discarded.

Notably, the concentrations of Csn-B selected for this study fall within a range that triggers monolayer expansion by more than 5%. Higher concentrations were not explored to remain within pharmacologically relevant proportions. The values of the surface compressional modulus show that Csn-B generally reduces this parameter for DPPE, making it less rigid. This is expected when some molecules are inserted into condensed monolayers^33−35^ since this promotes more possibility of molecular rearrangements other than dense packing. For DOPE, however, there is no perceptible change in the *K* values, which is related to a lower degree of elasticity of the pure lipid, which facilitates the drug incorporation with a less drastic effect on the film’s mechanical properties.

**Figure 3:**
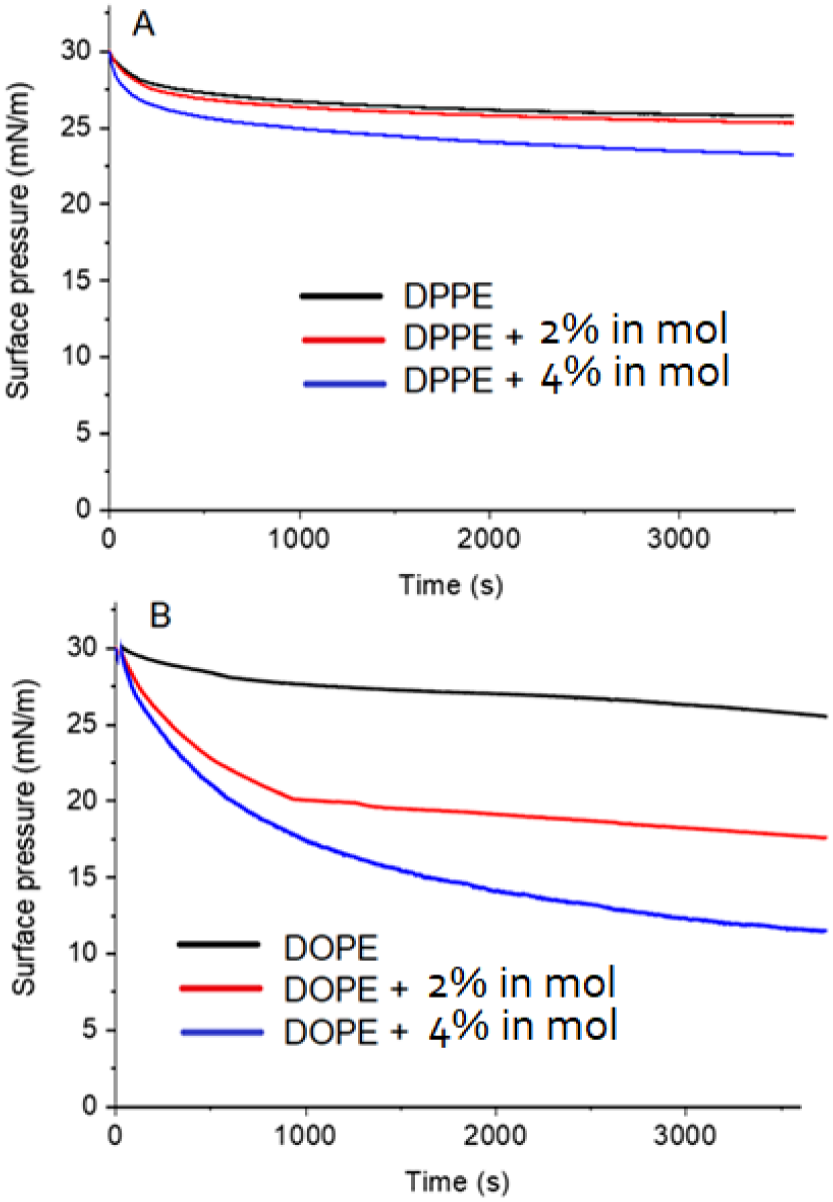
Surface pressure–time isotherms for DPPE (A) and DOPE (B) monolayers, alone or with Csn-B previously compressed to 30 mN/m at a constant area.

Figure 3 illustrates the stability of the monolayers as they undergo compression to 30 mN/m and subsequently relax. This specific surface pressure value was selected due to its reported correspondence with the mechanical characteristics of natural bilayers, particularly in terms of molecular density.^35−37^ Notably, the introduction of Csn-B destabilizes both monolayers, which is evident from the higher decay rate than that observed with pure lipids. However, the destabilizing effect is more pronounced in the case of DOPE. A hydrophobic compound like Csn-B can destabilize a Langmuir monolayer when compared to a pure lipid monolayer, as observed in relaxation experiments (surface pressure-time curves). This destabilization occurs for several reasons:

(i) Hydrophobic interactions and packing: introducing a hydrophobic compound disrupts the organized packing of the lipid monolayer. The water-averse compound tends to position itself within the monolayer, typically near the hydrophobic tails of the lipids. This interaction weakens the cohesive forces among the lipids, leading to defects and imperfections such as holes within the monolayer. As a result, the monolayer’s overall packing efficiency and stability are compromised, which can manifest as a sudden drop in surface pressure.

(ii) Overall destabilization: these combined effects result in a less stable, more loosely packed monolayer compared to a pure lipid system. Relaxation experiments reveal this destabilization through decreased surface pressure over time, suggesting a more flexible and easily deformable film.

In summary, this destabilization is more pronounced in more flexible lipids like POPE, which have a looser packing structure and are more susceptible to molecular rearrangements. In contrast, DPPE, which has a more tightly packed structure, exhibits greater resistance to these changes, allowing for long-term stability.

**Figure 4:**
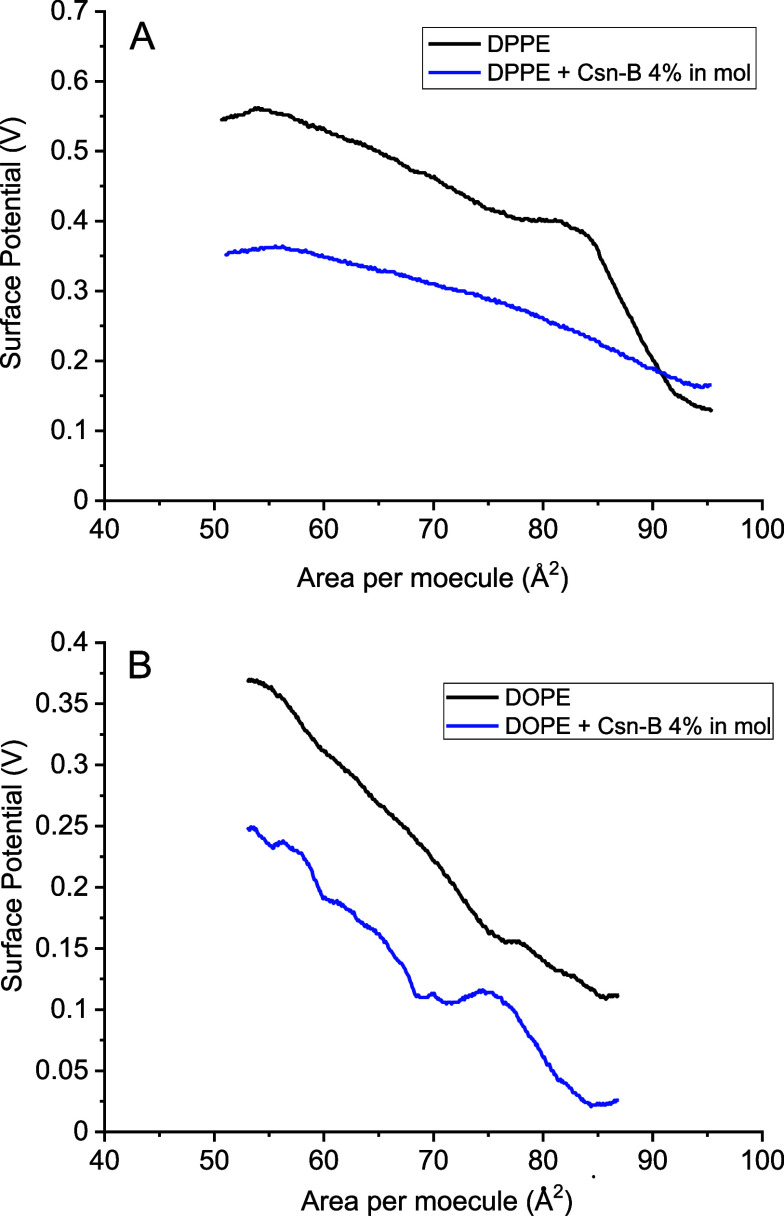
Surface potential–area isotherms for DPPE (A) and DOPE (B) monolayers, alone or with Csn-B.

This observation aligns with the surface potential data presented in Figure 4, where Csn-B induces a decrease in surface potential values for the lipids, signifying a diminished alignment of electrical dipoles concerning the surface normal.^38^

The surface potential of a phospholipid monolayer is subject to reduction owing to diverse factors. These encompass modifications in molecular structure, environmental conditions, and interactions with external molecules. The primary factor elucidated in our findings points to the molecular reorganization of lipid monolayers: shifts in the orientation and arrangement of phospholipid molecules at the air–water interface may bring about changes in molecular packing or orientation, thereby influencing the distribution of charge across the surface and resulting in a diminished surface potential.

The presence of shoulders in surface potential-area isotherms for Langmuir monolayers, such as those observed in pure DPPE and mixed Csn-B/DOPE monolayers, can be attributed to the impact of amphiphilic molecular packing and conformational changes at the air–water interface. As compression reduces the area per molecule, the molecules initially pack more closely, increasing surface potential due to dipole proximity. However, continuous compression may induce phase transitions and other molecular rearrangements, shifting the molecular organization from a loosely packed phase to a condensed phase, leading to plateaus or shoulders in the isotherm. Changes in molecular tilt angle may also influence the isotherm by altering vertical dipole orientation. Additionally, interactions between the drug and amphiphile in both regions, headgroups, and hydrophobic chains can affect the surface potential and packing efficiency. Strong repulsions may cause a sharper increase in surface potential, while weaker interactions may lead to less pronounced shoulders. The introduction of the drug typically decreases surface potential, impacting molecular inclination due to reduced lateral repulsions. Moreover, lipids with flexible chains, such as DOPE, may readily accommodate supramolecular structures, leading to more pronounced shoulders.

**Figure 5:**
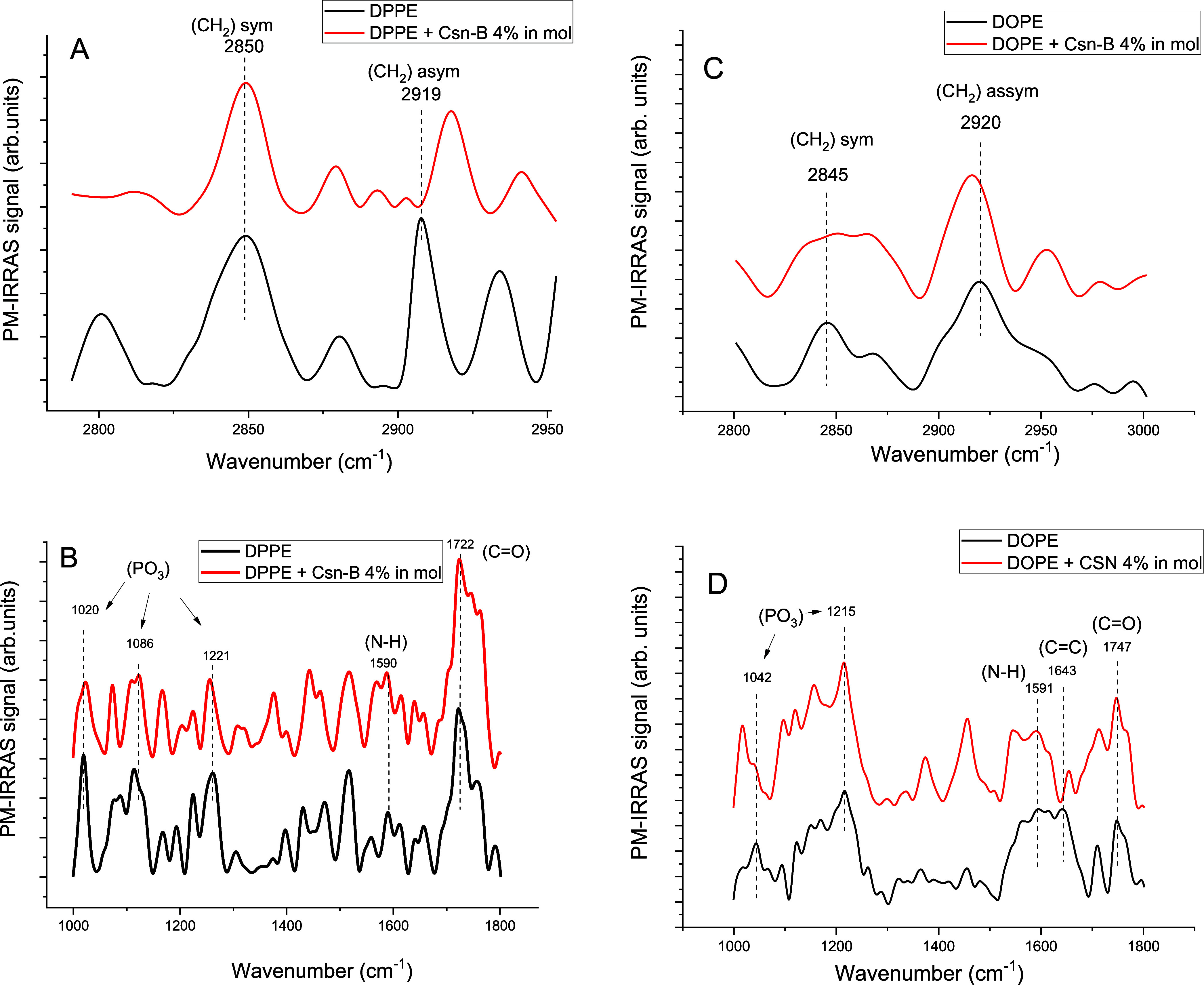
PM-IRRAS spectra for DPPE (A and B) and DOPE (C and D) monolayers, alone or with Csn-B at the surface pressure of 30 mN/m.

To explore the structural aspects of the monolayer more deeply, we conducted investigations utilizing PM-IRRAS, as showcased in Figure 5. Panels A and C focus on regions where the primary vibrational transitions of CH in the acyl chains occur. Generally, the band centered at approximately 2850 cm^–1^ corresponds to symmetric CH_2_ stretches, while the band at 2920 cm^–1^ relates to asymmetric CH_2_ stretches. Additional bands in this region pertain to CH_3_ stretches. The position of the CH_2_ asymmetric band (2919 cm^–1^ for DPPE) points to a very well-ordered monolayer of solid state, pointing to the all-trans conformation and a higher number of gauche conformations upon Csn-B incorporation due to the shift to high wavenumbers.

With the introduction of Csn-B, a noteworthy alteration in the overall spectral profile is observed, indicating that the drug interacts with and modulates the acyl chain order. The position of the CH_2_ groups and the ratios of asymmetric to symmetric intensities are typically associated with the all-trans/gauche conformer ratio.^39^ Although the effects of incorporating Csn-B on these parameters are not immediately evident, it is worth highlighting the broadening of the symmetric band in the DOPE monolayer, which could be indicative of monolayer fluidization.

Introducing hydrophobic compounds such as Csn-B into a lipid Langmuir monolayer can have varying effects on the all-trans/gauche ratio of the alkyl chains in the lipid, depending on several factors such as the interaction between the compound and the lipid, as well as the disruption of molecular packing. While favorable interactions between the hydrophobic compound and the all-trans conformation could increase the ratio by promoting a more ordered state through van der Waals forces, it is more common for hydrophobic compounds to disrupt the tight packing of lipid molecules. This disruption can introduce gauche kinks in the chains as they rearrange to accommodate the intruder, potentially decreasing the all-trans/gauche ratio.

The flexibility of the alkyl chains also plays a significant role. More flexible chains, such as those for DOPE, are naturally prone to adopting gauche conformations, even without the presence of a disruptive compound. Therefore, the shape and functionality of the introduced compound along the air–water interface can affect the outcome. Hydrophobic compounds that do not complement the all-trans conformation can promote a more disordered state through steric hindrance, pushing against the alkyl chains and forcing them to adopt gauche conformations. That is why the shift to a higher wavenumber, a primary indication of the higher numbers of gauche conformers, is noted for DPPE, a more ordered lipid, and, therefore, more susceptible to packing disturbance. For the DOPE, this effect can be better observed by broadening the bands.

Moreover, the inherent hydrophobicity of the compound can influence how deeply it inserts into the monolayer, causing more significant disruption and potentially greater gauche kink formation. Ultimately, the balance of these factors determines the final effect on the all-trans/gauche ratio. Strong interactions between the compound and all-trans conformation may increase the ratio, but the disruptive effect of introducing a hydrophobic compound more often leads to a decrease in the ratio.

In addition to the aforementioned alterations, the influence of Csn-B extends to the hydrophilic region, as demonstrated in panels B and D. This impact is particularly noticeable in the behavior of phosphate groups but less so in the carbonyl groups. The ester carbonyl stretching band corresponds to the stretching vibration of the C=O bond in the ester linkage of the phospholipid molecule. It typically appears at 1722 cm^–1^ for DPPE. In contrast, DOPE exhibits two bands for this vibration: 1710 and 1747 cm^–1^, with the lower wavenumber band reflecting the C=O group more exposed to hydrogen bonding interactions with neighboring molecules or water molecules. With Csn-B, these bands for both DPPE and DOPE show no significant change.

Distinctive features are observed regarding the phosphate stretching vibrations, which typically span the 1000 to 1200 cm^–1^ range. The higher wavenumber asymmetric stretching occurs around 1200 cm^–1^, while the symmetric stretching manifests at slightly lower wavenumbers, approximately 1080 cm^–1^. Within the presented spectra, primary bands are discernible at 1020, 1086, and 1122 cm^–1^ for DPPE, attributed respectively to phosphate bending, symmetric, and asymmetric stretching modes. The vibrational bands can vary depending on lipid composition, packing density, and intermolecular interactions within the monolayer. With Csn-B, the peaks exhibit no significant shift, but slight changes in shape and broadness are observed. Consequently, we note only subtle variations upon adding the drug. Considering the inherent variability in wavenumbers depending on the specific phospholipid and its environment, along with considerations of signal-to-noise ratios in the spectra, we cannot confidently assert notable changes in the polar groups attributed to interactions with the drug.

However, for DOPE, the two bands centered at 1042 and 1215 cm^–1^ are the two ones more distinguishable in the spectra, particularly regarding the baseline and surrounding bands and noises. Interestingly, the band centered at 1042 cm^–1^ shifts to lower wavenumbers upon Csn-B incorporation. This shift reflects that the less packed monolayer of DOPE allows the hydrophobic drug to interact more effectively with the polar heads. This interaction, likely due to molecular accommodation, may disrupt the adhesion of the polar groups to water, thereby reducing surface pressure and, consequently, thermodynamic stability, which is consistent with the results presented in the relaxation experiments.

The interactions between small hydrophobic antimicrobial drugs and lipid Langmuir monolayers have been recently investigated due to their valuable insights into the interactions between compounds like Csn-B and cell membranes.^40−44^ Key findings from research in this area relate to the drug partitioning within the monolayer. The degree of partitioning is influenced by factors such as the drug’s hydrophobicity and the characteristics of the lipid film, such as chain length and headgroup charge.^40,41^ Some studies suggest a potential correlation between membrane disruption and certain antimicrobial drugs, as they can disturb the integrity of the lipid monolayer, mirroring their effects on bacterial membranes. This disruption is often manifested as a decrease in surface pressure or alterations in the surface pressure-area isotherm of the monolayer.^42,43^ Additionally, the molecular properties of the drug, including size, charge, and functional groups, play a significant role in its interaction with the monolayer. For instance, cationic drugs may exhibit stronger interactions with negatively charged lipid headgroups.^41,43^

Studies involving PE lipids, such as those examining nystatin, violacein, and thymol, provide further insight.^44−46^ Nystatin, a broad-spectrum antibiotic, demonstrates relatively high hydrophobicity and has been observed partitioning into PE monolayers, potentially disrupting their organization and reducing surface pressure, thereby affecting membrane integrity.^44^ Sakuranetin, an antimicrobial drug, is another example of a small hydrophobic molecule known to interact with DPPE monolayers, possibly altering their packing density and fluidity and consequently impacting microbial membrane function.^45^ Polygodial, renowned for their antimicrobial properties owing to their hydrophobic nature, have been shown to disrupt DPPE monolayer structure and fluidity, potentially leading to membrane leakage.^46^

Although there are fewer studies specifically focusing on small hydrophobic drug interactions with DOPE monolayers compared to DPPE, certain trends have emerged. Some drugs may exhibit reduced interaction with DOPE compared to DPPE due to electrostatic repulsion between the positively charged drug and the DOPE headgroup.^47^ Generally, neutral hydrophobic drugs are expected to partition into DOPE monolayers compared to charged drugs more readily.

Regarding the distinction between saturated and unsaturated lipids, lisicamine has been found to have a stabilizing effect on unsaturated lipids compared to saturated ones with choline headgroups, with minimal impact on film elasticity. However, studies suggest that such drugs can disrupt both saturated and unsaturated monolayers, with potentially greater effectiveness in disrupting the more rigid saturated monolayers.^44−46^ In comparison to our previous studies using PS (phosphatidylserine) lipids (DPPE and DOPE),^29^ we can identify several similarities and differences that illuminate the effects of polar groups on the interaction with Csn-B. For both groups of lipids, we observed a decrease in surface potential and a reduction in the all-trans/gauche ratio. This suggests that the lipids become less orderly upon interaction with the drug. Additionally, for both groups, the surface compressional modulus decreases for the saturated lipids (DPPS and DPPE) in the presence of the drug. This decrease indicates that the monolayer becomes less mechanically sensitive to compression due to increased molecular flexibility and, therefore, lower stiffness. However, this effect was not noticeable for the unsaturated lipids, as their compressional modulus remained relatively unchanged with Csn-B. This stability is attributed to the high fluidity of the lipid monolayer in its pure form, typical of unsaturated lipids. Regarding the differences between PS and PE lipids, we noted that while the incorporation of the drug condensed the monolayer in DPPS (shifting it to lower areas at most given surface pressures), it expanded the monolayer in its unsaturated counterpart. Conversely, for PE lipids, the drug caused expansion in DPPE and condensation in the unsaturated counterpart. The main similarities can be attributed to the nature of the alkyl (hydrophobic) chains, whereas the differences are clearly related to interactions with the polar head groups. Since surface pressure is primarily a thermodynamic measure of intermolecular interactions at the interface with the aqueous bulk phase, this finding implies that the molecular interactions of Csn-B with lipids are influenced not only by the saturation level of the alkyl chains but also by the chemical nature of the polar head groups.

It is important to note that this initial analysis remains preliminary, and we anticipate that these findings will lay the groundwork for a more comprehensive molecular understanding in future investigations.

## 4 Conclusions

Distinct effects were observed on DPPE and DOPE monolayers at the air–water interface upon introducing Csn-B. Both lipids exhibited expansion at low surface pressures, indicating drug incorporation, but further compression prompted a molecular rearrangement of the drug at the interface. DPPE showed more substantial expansion, particularly at higher molecular areas. Ongoing compression led to monolayer condensation, attributed to cytosporone redistribution within lipid interstices mitigating lateral repulsion or expelling from the monolayer. This compression also reduced in-plane elasticity, with DPPE experiencing a more pronounced effect, suggesting the emergence of viscoelastic characteristics within the mixed structure. Notably, both monolayers displayed destabilization, evidenced by surface pressure-time and surface potential measurements. Infrared spectroscopy could further elucidate these effects, revealing interactions affecting hydrophobic and hydrophilic groups, with a higher effect on hydrophobic tails, notably increasing gauche conformers for DPPE, likely due to its initially more packed state and more significant impact with the introduction of a hydrophobic drug.

When comparing the effects of the drug on the two types of lipids-one with a fully saturated alkyl chain, providing a highly packed monolayer, and the other with a double bond providing a more superficially compressible monolayer—we observed some distinctions. It appears that Csn-B capitalizes on the lower rigidity of DOPE, allowing for a more significant interaction with the polar PE group. This effect likely stems from steric restrictions in the hydrophobic region and the subsequent re-accommodation of the drug in defects closer to the hydrophilic region of the monolayer. As a relatively small hydrophobic drug, Csn-B exerts a notable destabilizing effect by disrupting the adhesion of the phospholipid polar group to the aqueous interface, thereby destabilizing the monolayer. This effect is prominently evident across all data. However, it differs from DPPE in several key aspects: a shift toward smaller areas in the surface pressure-area isotherms, a more accelerated pressure drop in the relaxation experiments, and more significant effects on the polar regions in vibrational spectroscopy.

These findings hold significant implications for understanding the potential biological activity of Csn-B and its underlying molecular mechanisms when interacting with biological interfaces, such as cellular membranes and liposomes used in drug delivery applications. They also suggest distinct interaction mechanisms, particularly concerning antimicrobial action, compared to previously published findings on lipids commonly found in tumor cells, such as serine lipids.

## Supporting Information Available

Brewster angles microscopy images (Figure S1) for the monolayers (PDF)
