# Supplementary material for: Surface Chemistry of Cytosporone-B Incorporated in Models for Microbial Biomembranes as Langmuir Monolayers
Source: Langmuir. 2024 Jul 15;40(30):15749–57. doi: 10.1021/acs.langmuir.4c01575 (PMC11295194; doi:10.1021/acs.langmuir.4c01575)
Supplement: Supplementary file 1 — la4c01575_si_001.pdf [file la4c01575_si_001.pdf]

Supporting Information for Publication

Surface Chemistry of Cytosporone-B incorporated in models for microbial biomembranes as Langmuir Monolayers

Guilherme Nuñez Jaroque, Augusto Leonardo dos Santos, Patrícia Sartorelli, Luciano Caseli*

Department of Chemistry, Institute of Environmental, Chemical and Pharmaceutical Sciences, Federal University of São Paulo (Unifesp), Diadema, São Paulo, Brazil

* corresponding author: lcaseli@unifesp.br

1Figure

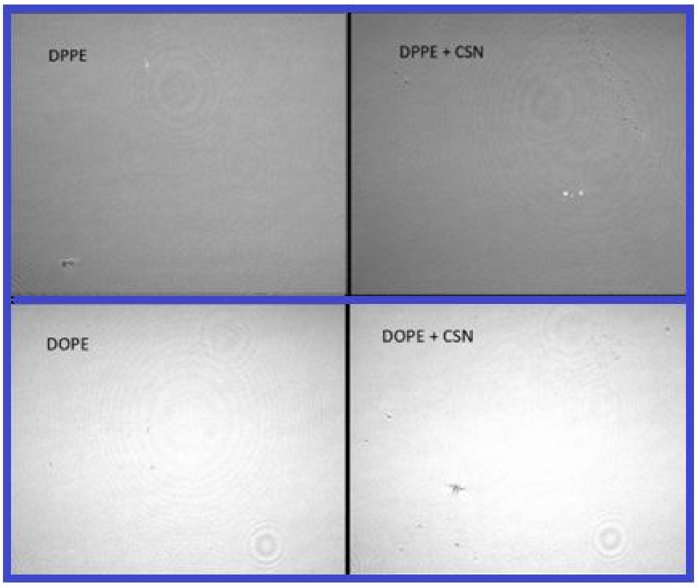

Figure S1: BAM images (3600 x 2400 μm) for DPPE and DOPE monolayers, alone or with Csn-B (0.5 mg/mL, 10 μL) at the surface pressure of 30 mN/m.
